# Dietary pattern modifies associations between dietary factors and pace of epigenetic aging

**DOI:** 10.3389/fnut.2026.1858850

**Published:** 2026-07-01

**Authors:** Deana M. Ferreri, Nanette V. Lopez, Jay T. Sutliffe, Chloe A. Sutliffe, Ryan Smith, Natalia Carreras-Gallo, Varun B. Dwaraka, Kirsten Seale, Joel H. Fuhrman

**Affiliations:** 1 Nutritional Research Foundation, Flemington, NJ, United States; 2 Department of Health Sciences and the PRANDIAL Lab, Northern Arizona University, Flagstaff, AZ, United States; 3 TruDiagnostic, Inc., Lexington, KY, United States

**Keywords:** biological aging, carotenoids, DNA methylation, epigenetic clocks, flavonoids, plant-based diet

## Abstract

**Introduction:**

Epigenetic clocks, DNA methylation-based biomarkers indicating biological aging, are responsive to diet and lifestyle changes, but it remains unclear how specific dietary factors affect these indicators. Identifying dietary factors and overall dietary patterns that influence epigenetic clocks could help to inform dietary recommendations for healthy aging. Plant food-derived dietary factors, such as carotenoids, folate, and flavonoids, have been associated with a lower risk of cancer, but there has been little research on their associations with epigenetic aging, in particular, the pace of aging indicator DunedinPACE. We previously reported a slower pace of epigenetic aging, indicated by DunedinPACE, in females following a nutrient-dense, plant-rich (“Nutritarian”) diet compared to those following a standard American diet (SAD). Here, in a secondary analysis, we aimed to determine whether selected food-derived dietary factors associated with reduced cancer risk and emphasized in the Nutritarian diet influenced DunedinPACE in this cohort.

**Methods:**

We investigated associations of carotenoids, flavonoids, and folate with DunedinPACE and other epigenetic aging indicators.

**Results:**

The Nutritarian group had significantly higher intakes of anthocyanidins, flavonols, flavan-3-ols, flavones, alpha-carotene, lutein and zeaxanthin, total folate, and food folate than the SAD group. With adjustment for diet group, body mass index (BMI), and menopausal status, intakes of anthocyanidins, flavonols, flavan-3-ols, lutein + zeaxanthin, total folate, and food folate were significantly associated with DunedinPACE. There were also significant dietary factor by group interactions. Higher intakes of these dietary factors were associated with lower DunedinPACE values in the Nutritarian group than in the SAD group.

**Conclusions:**

These results suggest that overall dietary patterns modify the relationships of individual food-derived nutrients and phytochemicals with DunedinPACE, and further support a Nutritarian diet as a strategy to slow epigenetic aging.

## 1 Introduction

Epigenetic clocks have been developed to indicate biological aging. First-generation clocks were trained to predict chronological age based on DNA methylation data (1, 2). Second-generation clocks, including GrimAge and PhenoAge, were trained to predict mortality, chronic disease, and other age-associated health outcomes (3, 4). DunedinPACE, a “third-generation” clock is unique in its use of longitudinal data, collected on 1,037 individuals of the same chronological age over 20 years. The pace of aging for each participant was developed using 19 biomarkers of cardiovascular, metabolic, liver, kidney, lung, immune, and periodontal health at ages 26, 32, 38, and 45. Biomarkers included BMI, HbA1C, blood pressure, VO_2_Max, lipid levels, white blood cell count, and C-reactive protein (CRP). The pace of aging was determined based on the rate of change of the 19 biomarkers, and a DNA methylation algorithm was developed as an indicator of the pace of aging (5–8). The different epigenetic clocks were developed using different sets of biomarkers, with limited overlap between clocks. For example, PhenoAge, GrimAge, and DunedinPACE all included CRP, PhenoAge and DunedinPACE both included white blood cell count, and GrimAge and DunedinPACE both included leptin (3, 4, 6).

The DunedinPACE pace of aging indicator has been associated with the intake of food groups, such as vegetables and fruits (negative association) and red and processed meats (positive association) (9, 10). An analysis of interventional studies found that second- and third-generation clocks were responsive to diet and lifestyle interventions, with the strongest responses observed in DunedinPACE compared to other clocks. Reductions in DunedinPACE were observed following calorie restriction, a vegan diet, a Mediterranean diet, and a Green Mediterranean diet (9). The Mediterranean and Green Mediterranean diets in particular aimed to increase total polyphenol intake (11). On the other hand, a dietary supplement that included vitamin B12, vitamin C, zinc, selenium, levomefolic acid (L-methylfolate), and botanical extracts did not improve DunedinPACE (12). Thus, how specific food-derived nutrients or phytochemicals influence DunedinPACE is unclear.

In the Women’s Health Initiative (WHI), intakes of the soy phytoestrogen coumestrol and beta-carotene were associated with slower epigenetic age acceleration (EAA) based on PhenoAge. In contrast, added sugars and (pre-formed) vitamin A were associated with faster EAA (13). Biomarkers of carotenoid intake have been associated with slower EAA based on the Hannum, GrimAge, and PhenoAge clocks (3, 4, 14). Plasma folate was associated with lower GrimAge and PhenoAge in a study of veterans and food folate with younger biological age in NHANES participants (15, 16). Low vitamin C intake was associated with faster EAA rates, as measured by the Hannum and Horvath clocks (17). However, as of yet, there is scarce data on which individual nutrients and phytochemicals influence epigenetic aging, especially DunedinPACE (18). A secondary analysis of the Methylation Diet and Lifestyle study, an eight-week randomized controlled trial, found that intake of “methyl adaptogens,” a category including turmeric, garlic, berries, green and oolong teas, and rosemary, was associated with slower EAA based on the Horvath clock after adjusting for weight change during the intervention, but did not investigate second-generation clocks or DunedinPACE (19). However, a 12-month nutraceutical intervention including vitamin B12, vitamin C, zinc, selenium, levomefolic acid, and botanical extracts showed varying effects on epigenetic aging indicators, and notably increased DunedinPACE, suggesting an increase in the pace of aging (12).

Diet and other environmental exposures affect health outcomes in part via epigenetic modifications such as DNA methylation. DNA methylation itself is dependent on the one-carbon metabolism pathway which requires micronutrients including folate as cofactors or precursors. Diet can affect DNA methylation by altering the availability of micronutrient cofactors, the activity of enzymes involved in one-carbon metabolism, or the activity of DNA methyltransferases (20). Intakes of antioxidant vitamins C and E are associated with altered DNA methylation patterns, according to epigenome-wide association studies, and dietary phytochemicals including flavonoids have been shown to modulate activity of DNA methyltransferases (21–24). Patterns of DNA methylation change with aging and alterations in methylation patterns are associated with chronic diseases including cancers. For example, hypermethylation in the promoter regions of tumor suppressor genes or hypomethylation of oncogenes increases cancer risk. Previous studies have associated epigenetic age acceleration with cancer risk using GrimAge, PhenoAge, and DunedinPoAm (an earlier version of DunedinPACE) (25–30). There is limited data on the relationship between DunedinPACE and cancer risk. A prospective study of U. S. veterans found faster pace of aging indicated by DunedinPACE was associated with a higher risk of developing cancer over the average 13-year follow-up period (31). The extent of overlap between methylation patterns at CpG sites responsive to dietary factors, those involved in the development of cancer, and those that epigenetic clocks measure is not yet clear.

A nutrient-dense, plant-rich (NDPR) or Nutritarian diet is a plant-based diet that emphasizes cruciferous vegetables, beans and legumes, the onion and garlic family, mushrooms, berries, nuts, and seeds, while minimizing animal foods, oils, and refined carbohydrates. Nutritarian dietary recommendations are based on the literature, particularly on the potential cancer-preventive actions of foods and food-derived phytochemicals. Higher intakes of vitamins, minerals, and plant food-derived compounds, such as anthocyanidins, carotenoids, and soy isoflavones, have been associated with reduced cancer risk (32–36). Carotenoids, such as alpha- and beta-carotene, lycopene, lutein, and zeaxanthin, are found in green, yellow/orange, and red vegetables and fruits; flavonoids are found in tea, apples, berries, grapes, citrus fruits, onions, kale, broccoli, and soybeans; and food folate, a B-vitamin is found in green vegetables and legumes (35–40).

We recruited females following either a Nutritarian diet or a standard American diet (SAD), and previously compared dietary inflammatory potential, epigenetic age indicators, and other epigenetic biomarkers in these two populations. The pace of epigenetic aging, as indicated by DunedinPACE, was significantly slower in the Nutritarian group. However, there were no significant differences between groups in EAA indicators derived from Horvath, Hannum, GrimAge, and PhenoAge clocks. Lower dietary inflammatory potential and epigenetic markers indicating a lower inflammatory status were observed in the Nutritarian group. Specifically, the Nutritarian group showed differences in methylation-predicted immune cell populations (lower neutrophils and higher T regulatory cells) and a lower epigenetic biomarker proxy (EBP) for C-reactive protein (CRP). An EBP for blood glucose was lower in the Nutritarian group (41).

Here, we investigated how specific dietary factors related to cancer prevention influenced epigenetic aging in females following either a standard American diet or a plant-based diet emphasizing these dietary factors. We analyzed nutritional data from this population to investigate associations between food-derived dietary factors and the pace-of-aging indicator, DunedinPACE, and epigenetic age acceleration based on the PhenoAge and GrimAge epigenetic clocks (principal component-based). To further study the impact of these dietary factors on epigenetic aging in a larger population, we included the TruDiagnostic Biobank cohort of 28,030 participants who had completed TruDiagnostic’s commercial TruAge test. In this cohort, we examined associations of DNA methylation-predicted vitamin and carotenoid status with epigenetic clocks.

## 2 Methods

This study was approved by the Institute of Regenerative and Cellular Medicine Institutional Review Board (IRCM-IRB) and was conducted in compliance with the Belmont Report and Good Clinical Practice Guidelines. IRB: IRCM-2022-335. Informed consent was collected using REDCap (42). All participants received their personal epigenetic aging test results after their involvement in the study. All shared personal information was de-identified.

### 2.1 Study population

As previously described, females aged 40–75 years were recruited and screened for a cross-sectional study conducted from 2023–2024 with retrospective self-reporting of dietary patterns, which investigated inflammatory biomarkers, dietary inflammatory status, and epigenetic aging in women following a Nutritarian or standard American diet (41). Briefly, 47 non-pregnant female participants habitually following a nutrient-dense, plant-rich (NDPR) or Nutritarian diet for at least 5 years, and 49 females habitually following a standard American diet (SAD) for at least 5 years were recruited. Additional inclusionary criteria for the SAD group included not currently or within the past 2 years following any weight loss plan, BMI < 30.0, and consuming a maximum of 8 alcoholic drinks per week. Additional inclusionary criteria for the Nutritarian group included a BMI ≤23 for at least 2 years and a maximum of two alcoholic beverages per week. The Nutritarian BMI criterion was based on the BMI of women on long-term plant-based diets as reported from EPIC-Oxford and the Swedish Mammography Cohort (43, 44).

The TruDiagnostic Biobank cohort includes 28,030 participants (59% male), recruited primarily from the United States between October 2020 and November 2025, who completed the commercial TruDiagnostic TruAge test and had their DNA methylation data generated. The majority of the samples were collected under a healthcare provider’s recommendation or guidance, while less than 5% were collected in a direct-to-consumer setting, potentially introducing self-selection bias, as most participants were likely to seek preventive care and had fewer comorbidities than typical patient populations. Participants were also asked to complete a survey about personal information, medical history, social history, lifestyle, and family history.

### 2.2 Demographic and dietary data

Participants reported health and demographic information, including age, sex, height, weight, medical history, and caffeine and alcohol use. Data were collected and managed using REDCap, an electronic data capture tool hosted by the Nutritional Research Foundation (45–47). REDCap (Research Electronic Data Capture) is a secure, web-based software platform designed to support data capture for research studies, providing (1) an intuitive interface for validated data capture; (2) audit trails for tracking data manipulation and export procedures; (3) automated export procedures for seamless data downloads to standard statistical packages; and (4) procedures for data integration and interoperability with external sources.

Nutrient intake was assessed using the Arizona Food Frequency Questionnaire (AFFQ), a semi-quantitative 175-item food frequency questionnaire in which respondents reported how often they usually consume each food and whether their usual portion size was small, medium, or large. The AFFQ computes values for 161 nutrients and 86 other derived variables from a database of more than 800 foods. Intakes of vitamins, carotenoids, and flavonoids were computed by the AFFQ. Data on the use of multivitamins and other dietary supplements were also collected in the AFFQ (48, 49).

### 2.3 DNA methylation assessment

Participants were shipped a test kit containing a lancet, a blood spot card, and a mailer for returning the sample to TruDiagnostic. DNA extraction was performed, and 500 ng of DNA was subjected to bisulfite conversion using the EZ DNA Methylation kit from Zymo Research, following the manufacturer’s protocol. The bisulfite-converted DNA samples were then randomly allocated to designated wells on the Infinium HumanMethylationEPIC BeadChip or the Infinium HumanMethylationEPICv2 BeadChip. The samples were amplified, hybridized onto the array, and subsequently stained. After the washing steps, the array was imaged using the Illumina iScan SQ instrument to capture raw image intensities, enabling further analysis.

The *Minfi* R package was used to preprocess DNA methylation (DNAm) data (50). In the sample quality control, we did not identify any samples with aberrant methylation levels or background signal levels (mean *p*-value higher than 0.05).

### 2.4 Statistical analyses and reproducibility

#### 2.4.1 DNA methylation clocks and epigenetic measures

We used DNAm data to calculate a series of measures commonly referred to as epigenetic clocks. We computed four clocks designed to predict the chronological age of the donor: Horvath Pan Tissue (1), Horvath Skin and Blood (1), and Hannum (2); three clocks designed to predict mortality, DNAmPhenoAge (50), GrimAge (51), and OMICmAge (52); a clock to measure telomere length, DNAmTL (53); and a DNAm measure of the rate of deterioration in physiological integrity, the DundedinPACE (6).

Non-principal component-based (non-PC) Horvath, Hannum, and DNAmPhenoAge epigenetic metrics were calculated using the methyAge function in the *ENMix* R package (54). To calculate the principal component-based epigenetic clock for the Horvath multi-tissue clock, Hannum clock, DNAmPhenoAge clock, GrimAge clock, and telomere length, we used the custom R script available via GitHub.^1^ The pace of the aging clock, DunedinPACE, was calculated using the PACEProjector function from the DunedinPACE package available via GitHub.^2^ SystemsAge was derived by projecting DNAm profiles onto a pre-trained PCA, aggregating principal components into organ-system–specific scores, and scaling the resulting composite age relative to chronological age using a reference cohort.

To calculate the epigenetic age acceleration (EAA) of the age-based clocks (except DunedinPACE, which indicates the pace of aging), we fit a regression model relating individuals’ chronological age to the different epigenetic age measures. We also included the array type and BMI to control for potential batch effects and differences due to BMI. Independent samples *t*-tests were performed between groups at a significance level of *p*-value below 0.05. Most of the metrics met the assumptions of the *t*-test, as the samples were independent. We evaluated normality using the Shapiro–Wilk test and homogeneity of variance using Bartlett’s test.

Epigenetic biomarker proxies (EBPs) for plasma metabolites were estimated with DNA methylation using models previously developed by Carreras-Gallo et al. Briefly, data on plasma protein and metabolite levels and clinical variables were collected from blood samples and electronic medical records from participants in the Massachusetts General Brigham (MGB) Biobank and matched to methylation data. An elastic net regression model was used to generate epigenetic biomarker proxies for each variable. EBPs were selected to estimate the protein, metabolite, or clinical variable if they had a significant (*p* < 0.05) Pearson correlation above 0.2 with that variable (55). EBPs for multiple carotene diols (surrogate markers of carotenoid status) and B vitamins are reported. Carotene diols 1, 2, and 3 represent three distinct groups of carotenoid isomers and metabolites detected in plasma, each with similar mass spectrometry features. EBPs were evaluated in the study population and TruDiagnostic’s cohort.

#### 2.4.2 Diet, lifestyle, and demographic data

Analysis of continuous variables (e.g., age and physical, psychosocial, dietary, and activity characteristics) included calculating means and standard deviations. At the same time, frequencies and percentages were determined for categorical variables (e.g., race, ethnicity, marital status, and education level). To approximate a normal distribution, outliers more than 2.5 standard deviations from the mean were removed. Independent samples *t*-tests were used to analyze the differences in outcomes between the two diet groups (i.e., the Nutritarian and Standard American Diet (SAD) groups). Bivariate correlations were used to evaluate the associations among the epigenetic clocks. Regression analyses were performed to determine the associations between nutrients and epigenetic aging, including group and group-by-predictor interactions. Initial models did not include covariates, while subsequent analyses included total caloric intake as a covariate. Final models included BMI and menopausal status as covariates. IBM SPSS Statistics, version 31 (Armonk, NY), was used to analyze all diet, lifestyle, and demographic data.

## 3 Results

### 3.1 Participant characteristics

Our total sample included 98 female, non-smoking participants aged 40–75. Of 48 who habitually (≥5 years) followed a Nutritarian diet and 49 who habitually (≥5 years) followed a SAD, 47 in the Nutritarian diet group and 48 in the SAD group completed all questionnaires and epigenetic aging blood test kits. Participant characteristics were reported previously: age, race/ethnicity, education level, energy intake, and physical activity did not significantly differ between groups. However, a significantly higher proportion of the Nutritarian group was postmenopausal. Once outliers were removed for this secondary analysis, 44 participants remained in the SAD group, and 42 remained in the Nutritarian diet group. Age (59.5 in the Nutritarian group vs. 56.3 in the SAD group, *p* = 0.09) and education level did not differ between groups. Menopausal status remained significantly different, with 58.5% of the Nutritarian group and 11.6% of the SAD group being postmenopausal. Most participants were white (92.7% in the Nutritarian group and 83.7% in the SAD group), with no significant differences in race or ethnicity between groups. Differences between groups in body weight and BMI were expected based on the inclusion criteria, and significant differences were observed: lower body weight (54.86 kg vs. 69.68 kg, *p* < 0.001) and lower BMI (20.50 vs. 25.60, *p* < 0.001) in the Nutritarian group compared to the SAD group. Physical activity was non-significantly higher (9927.9 vs. 5350.1 total MET-minutes/week, *p* = 0.18) and sedentary time non-significantly lower (400.1 vs. 541.6 min/day, *p* = 0.09) in the Nutritarian group. In contrast to our previous report, after removing outliers, total energy intake was significantly different between groups (1,902 kcal in the SAD vs. 1,548 kcal in the Nutritarian group, *p* = 0.04).

### 3.2 Intakes of vitamins, flavonoids, and carotenoids

We selected several dietary factors emphasized in the Nutritarian diet and previously associated with reduced cancer risk to evaluate: flavonoid subclasses anthocyanidins, flavonols, flavan-3-ols, flavanones, flavones, total isoflavones, and the soy isoflavone genistein; carotenoids alpha-carotene and lutein + zeaxanthin; vitamin E; and total and food folate. Among flavonoids, a significantly higher intake in the Nutritarian group was observed for anthocyanidins, flavonols, flavan-3-ols, and flavones, but not flavanones or isoflavones. Significant differences were also observed between groups in the intake of alpha-carotene, lutein and zeaxanthin, total folate, and food folate (Table 1).

**Table 1:** Mean intakes with standard deviations of dietary factors in nutritarian (*n* = 42) and standard American diet (*n* = 44) groups.

Dietary factor	Mean (SD)		*p*-value
	SAD	Nutritarian	
Anthocyanidins (mg)	81.01 (108.97)	183.31 (121.17)	< 0.001
Flavonols (mg)	32.06 (32.07)	87.02 (49.52)	< 0.001
Flavan-3-ols (mg)	82.39 (139.36)	158.51 (170.10)	0.013
Flavanones (mg)	31.35 (41.43)	42.86 (46.10)	0.113
Flavones (mg)	2.31 (2.29)	4.38 (3.49)	<0.001
Isoflavones (mg)	4.04 (7.93)	6.10 (7.32)	0.108
Genistein (mg)	1.90 (3.76)	3.02 (3.65)	0.082
Alpha-carotene (mcg)	713.80 (750.61)	1882.67 (1847.30)	< 0.001
Lutein + zeaxanthin (mcg)	4304.48 (6633.44)	11526.74(9472.03)	< 0.001
Vitamin E (alpha-tocopherol, mg)	11.16 (10.38)	14.36 (6.56)	0.047
Total folate (mcg)	444.56 (332.16)	694.71 (340.08)	< 0.001
Food folate (mcg)	331.59 (243.29)	656.88 (344.20)	< 0.001

One-sided *p*-value (*t*-test).

**Figure 1:**
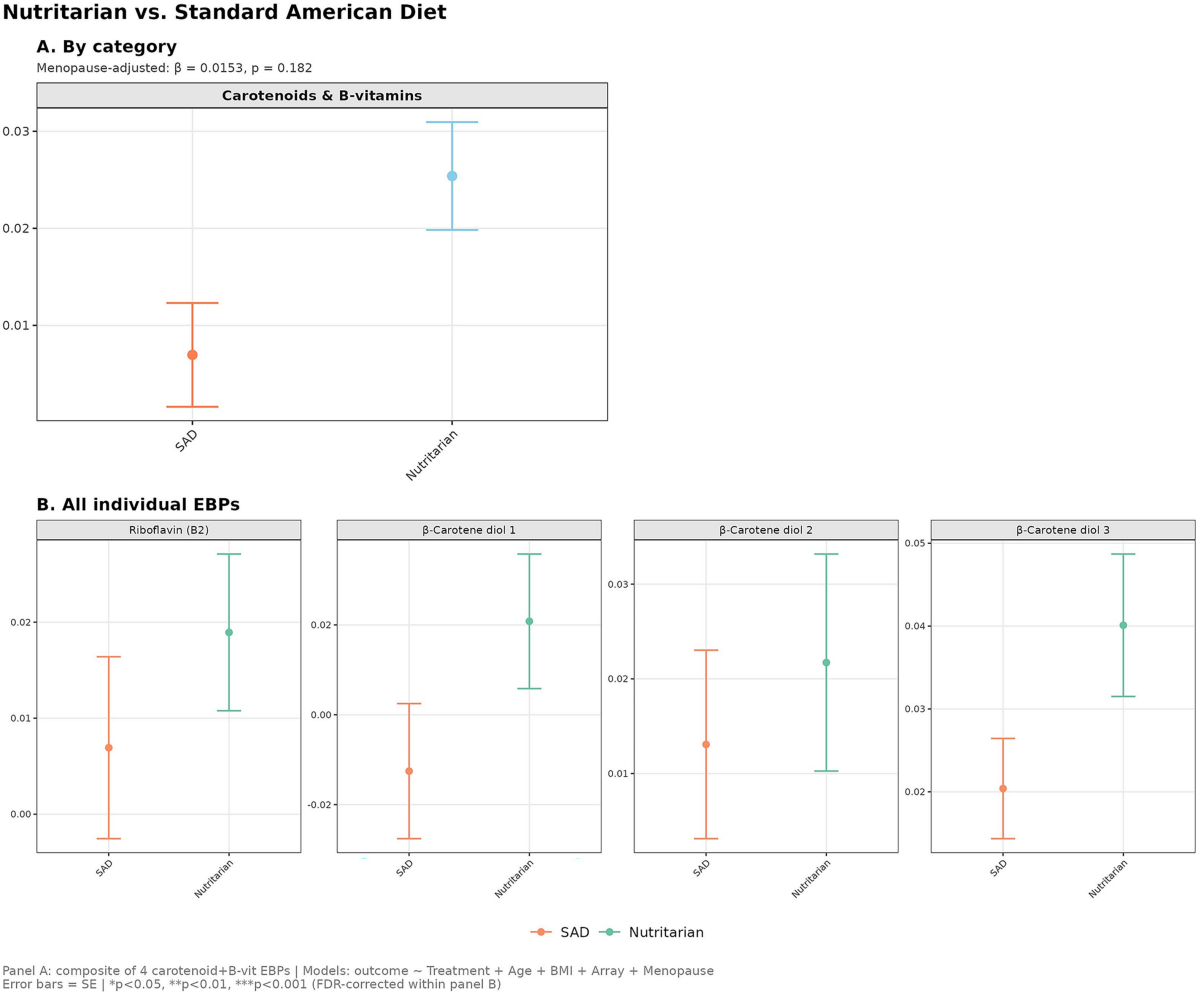
Epigenetic biomarker proxies (EBPs) of carotenoids and B vitamins. Epigenetic biomarker proxies (EBPs) estimating carotenoid and B-vitamin status are non-significantly higher in the Nutritarian group than the standard American diet (SAD) group. Plots show the mean values of EBPs with error bars representing standard error (SE). **(A)** EBP category including carotenoids and B-vitamins. **(B)** Individual EBPs: Riboflavin, β-carotene diol 1, β-carotene diol 2, and β-carotene diol 3. Statistical significance is denoted by *p < 0.05, **p < 0.01, and ***p < 0.001. EBP estimates were obtained from linear regression models including group, age, array, BMI, and menopausal status as covariates. Alt text: A plot showing mean plus and minus standard error of epigenetic biomarker proxies estimating carotenoid and B-vitamin status in the Nutritarian and standard American diet groups.

Among the dietary factor categories we evaluated, epigenetic biomarker proxies (EBPs) had been developed for carotenoids and B vitamins. Based on DNA methylation data, the EBPs for carotenoids and B vitamins were non-significantly higher in the Nutritarian group, consistent with the higher carotenoid intakes derived from the food frequency questionnaire. Individual EBPs corresponding to carotenoid and riboflavin status similarly showed non-significantly higher levels in the Nutritarian group (Figure 1).

### 3.3 Relationships between dietary factors and epigenetic aging indicators

Bivariate correlations were used to determine associations among the three epigenetic aging clocks. Although a significant correlation was found between PhenoAge EAA and GrimAge EAA, neither was significantly correlated with DunedinPACE (Table 2).

**Table 2:** Correlations between epigenetic aging indicators.

Epigenetic aging indicator	PhenoAgeAccel	GrimAgeAccel	DunedinPACE
PhenoAgeAccel			
GrimAgeAccel	0.542 (0.371, 0.6780; *p* < 0.001)		
DunedinPACE	0.030 (−0.186, 0.242 *p* = 0.789)	0.143 (−0.073, 0.347; *p* = 0.193)	

Pearson’s correlation coefficient (95% confidence interval; *p*-value).

**Figure 2:**
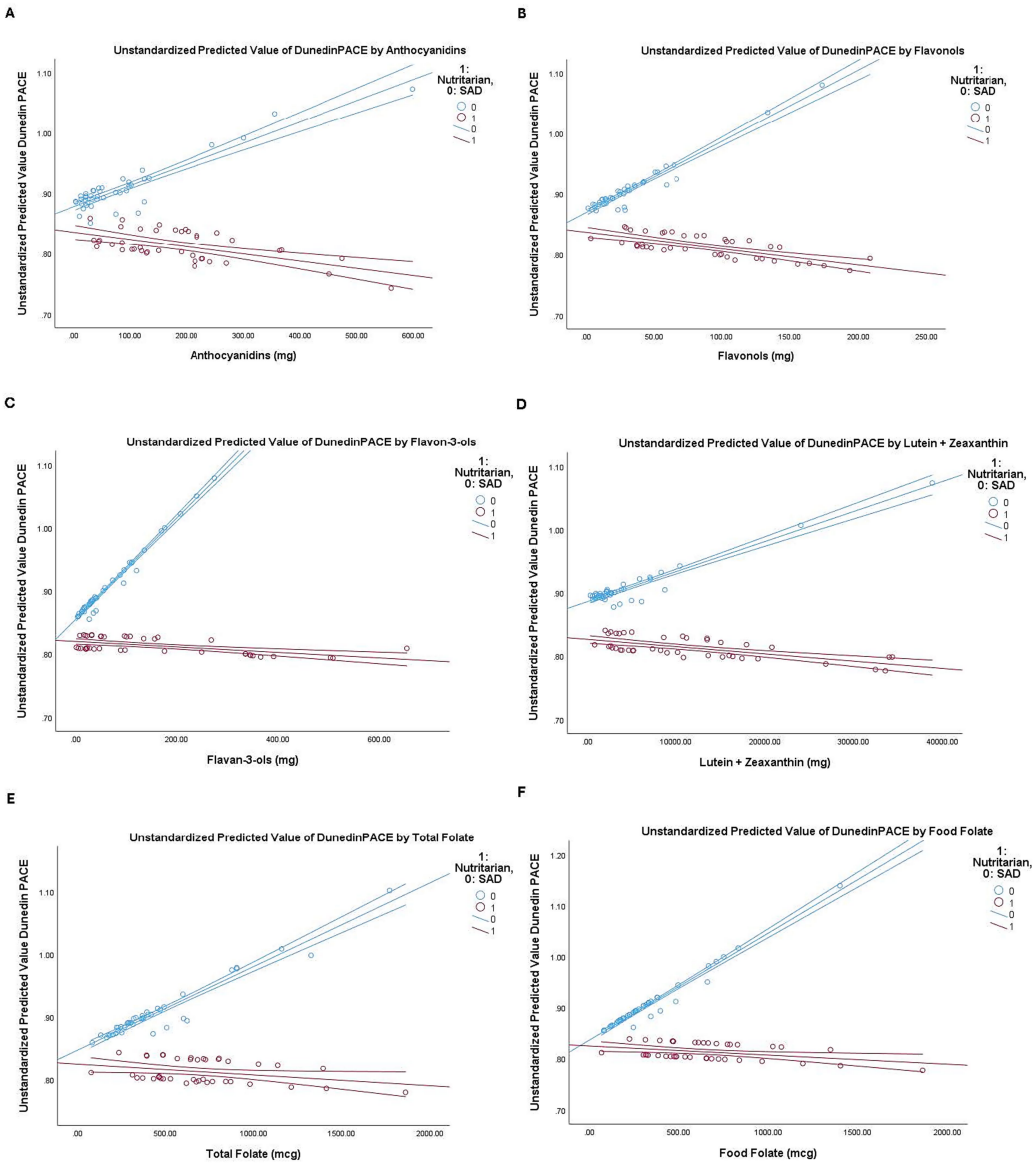
Associations between dietary factors and unstandardized predicted values of DunedinPACE by diet group. Associations between dietary factors and DunedinPACE by group in cases where dietary factor by group interaction was significant. Associations between dietary factors (x-axes) and DunedinPACE (y-axes) were assessed with a multiple regression model using diet group, BMI, and menopausal status as covariates. Each plot shows predicted DunedinPACE value and 95% confidence interval based on intake of each dietary factor. **(A)** Anthocyanidins, **(B)** Flavonols, **(C)** Flavan-3-ols, **(D)** Lutein + zeaxanthin, **(E)** Total folate, **(F)** Food folate. Alt text: Four scatterplots with trendlines showing intake of dietary factors on the x-axes (anthocyanidins, flavonols, flavan-3-ols, and food folate) and the predicted DunedinPACE values on the y-axes, based on multiple regression models using diet group (Nutritarian or standard American diet) and total energy intake as covariates.

We used linear regression models to examine the associations of each dietary factor with significantly different intakes between groups with second- and third-generation epigenetic clocks: epigenetic age acceleration (EAA) based on PhenoAge and GrimAge, and the pace of aging indicated by DunedinPACE. After adjusting for diet group, BMI, and menopausal status, we found no significant associations between dietary factors and EAA based on PhenoAge or GrimAge (data not shown). Intakes of anthocyanidins, flavonols, flavan-3-ols, lutein+zeaxanthin, total folate, and food folate were significantly positively associated with DunedinPACE. In addition, there were significant dietary factor by group interactions for each of these dietary factors (Table 3). Significant interactions are shown in Figure 2.

**Table 3:** Associations between dietary factors and DunedinPACE.

Variable	Overall F	*R* ^2^	Adj R^2^	Unstd β	Std β	*p*-value	95% CI
Anthocyanidins (mg)	*F*(5,77) = 6.68, *p* < 0.001	0.303	0.257	0.00039	0.463	0.004	0.00013, 0.001
BMI				0.003	0.107	0.494	−0.006, 0.013
Menopause				−0.037	−0.167	0.153	−0.088, 0.014
Group				−0.002	−0.012	0.952	−0.082, 0.077
Group × Anthocyanidins				−0.001	−0.618	0.006	−0.001, −0.00015
Flavonols (mg)	*F*(5,77) = 6.04, *p* < 0.001	0.282	0.235	0.001	0.554	0.013	0.00026, 0.002
BMI				0.001	0.043	0.788	−0.009, 0.011
Menopause				−0.025	−0.112	0.329	−0.075, 0.025
Group				−0.015	−0.070	0.722	−0.097, 0.067
Group × Flavonols				−0.001	−0.755	0.012	−0.003, −0.00033
Flavan-3-ols (mg)	*F*(5,77) = 7.72, *p* < 0.001	0.334	0.291	0.001	1.011	<0.001	0.00037, 0.001
BMI				0.001	0.021	0.892	−0.009, 0.010
Menopause				−0.020	−0.092	0.408	−0.069, 0.028
Group				−0.024	−0.115	0.479	−0.092, 0.044
Group × flavan-3-ols				−0.001	−1.101	<0.001	−0.001, −0.00036
Flavones (mg)	*F*(5,77) = 4.82, *p* < 0.001	0.238	0.189	0.009	0.271	0.160	−0.004, 0.022
BMI				0.004	0.128	0.427	−0.006, 0.014
Menopause				−0.017	−0.078	0.508	−0.069, 0.034
Group				−0.032	−0.151	0.422	−0.110, 0.047
Group × flavones				−0.012	−0.368	0.136	−0.027, 0.004
Alpha-carotene (mcg)	*F*(5,77) = 4.97, *p* < 0.001	0.244	0.195	0.000029	0.419	0.161	−0.000012, 0.00007
BMI				0.002	0.063	0.707	−0.009, 0.013
Menopause				−0.022	−0.099	0.399	−0.073, 0.030
Group				−0.036	−0.173	0.322	−0.109, 0.036
Group × Alpha-carotene				−0.000037	−0.568	0.100	−0.00008, 0.000007
Lutein + zeaxanthin (mcg)	*F*(5,77) = 5.38, *p* < 0.001	0.259	0.211	0.0000046	0.391	0.046	0.00000009, 0.000009
BMI				0.001	0.047	0.779	−0.009, 0.012
Menopause				−0.023	−0.105	0.369	−0.074, 0.028
Group				−0.042	−0.199	0.256	−0.115, 0.031
Group × Lutein + zeaxanthin				−0.0000058	−0.480	0.045	−0.000011, −0.00000014
Total folate (mcg)	*F*(5,77) = 6.90, *p* < 0.001	0.309	0.264	0.00014	0.477	0.002	0.000054, 0.00023
BMI				0.001	0.041	0.794	−0.009, 0.011
Menopause				−0.034	−0.153	0.182	−0.084, 0.016
Group				0.004	0.017	0.938	−0.086, 0.093
Group × total folate				−0.00016	−0.628	0.012	−0.00028, −0.000035
Food folate (mcg)	*F*(5,77) = 7.67, *p* < 0.001	0.332	0.289	0.00022	0.690	<0.001	0.000098, 0.00033
BMI				−0.00017	−0.005	0.973	−0.010, 0.010
Menopause				−0.030	−0.135	0.224	−0.079, 0.019
Group				0.005	0.023	0.909	−0.080, 0.090
Group × food folate				−0.00024	−0.908	0.002	−0.00038, −0.000090

**Figure 3:**
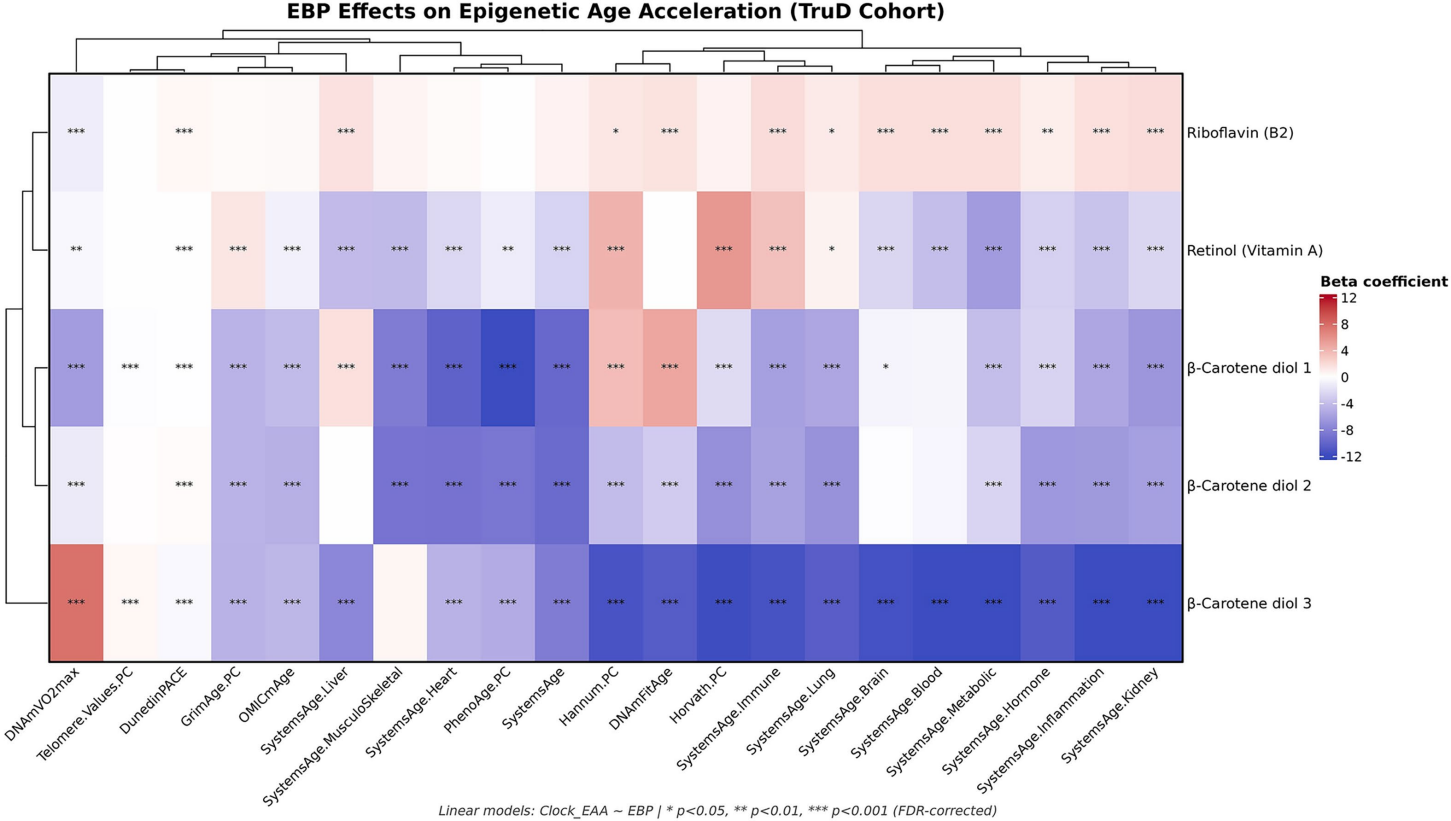
Heatmap of linear model effects of B-vitamin and carotenoid EBPs on epigenetic age acceleration indicators. Associations between epigenetic biomarker proxies (EBPs) estimating vitamin or carotenoid status and epigenetic age metrics. Heatmap shows beta-coefficients describing the associations between EBPs representing dietary factors and epigenetic age metrics from linear regression models. Statistical significance is denoted by *p < 0.05, **p < 0.01, and ***p < 0.001 (FDR corrected). EAA indicators were estimated from linear regression models including age, sex, and array as covariates. Alt text: A heatmap that shows beta-coefficient values ranging from −12 to +12 describing the associations between EBPs and multiple epigenetic aging metrics. The EBPs represent dietary factors riboflavin, retinol, and β-carotene diol 1, 2, and 3, and epigenetic aging metrics include DunedinPACE, GrimAge, and PhenoAge.

Our ability to detect a significant association between nutrient intake and DunedinPACE was likely limited by both small sample size and self-reporting of dietary intake. To examine the relationship between carotenoid status and epigenetic aging in a larger cohort and using objective nutrient intake markers, we evaluated EBPs estimating carotenoid status in 28,030 individuals from TruDiagnostic’s cohort (Figure 3). Associations between alpha-carotene intake and epigenetic clocks were not significant in our cohort of females following a SAD or Nutritarian diet, however, the association between lutein + zeaxanthin intake and DunedinPACE, but not PhenoAge EAA or GrimAge EAA, was significant. In comparison, in the TruDiagnostic cohort, higher methylation-predicted carotenoid levels were significantly associated with DunedinPACE, epigenetic age acceleration based on PhenoAge and SystemsAge, and system-specific blood, metabolic, and heart SystemsAge scores. The directions of these associations varied by carotene diol: diol 3 was primarily negatively associated with these epigenetic aging metrics, and diols 1 and 2 showed mostly negative, but some positive, associations.

## 4 Discussion

### 4.1 Influence of dietary patterns on the associations of vitamins, carotenoids, and flavonoids with DunedinPACE

In our previous analysis of this cohort, we found that the pace of biological aging, as indicated by DunedinPACE, was significantly slower in the Nutritarian group than in the SAD group (41). In this secondary analysis, we studied the effects of the self-reported dietary pattern group on the relationship of intake of selected nutrients and food-derived dietary factors with DunedinPACE. Adjusting for BMI and menopausal status, anthocyanidins, flavonols, flavan-3-ols, lutein + zeaxanthin, total folate, and food folate were significantly positively associated with DunedinPACE. Significant dietary factor-by-group interactions were observed for anthocyanidins, flavonols, flavan-3-ols, lutein + zeaxanthin, total folate, and food folate, suggesting that the overall dietary pattern influenced the associations between these dietary factors and DunedinPACE.

Intake of anthocyanidins, flavonols, flavan-3-ols, flavones, alpha-carotene, lutein+zeaxanthin, total folate, and food folate were significantly higher in the Nutritarian group. The EBP(s) for carotenoids were also non-significantly higher in the Nutritarian group (Figure 1). These differences were expected based on the definitions of the two dietary patterns. For example, green vegetables, which are emphasized in Nutritarian diet guidelines, are rich in lutein (56). Anthocyanidins are primarily found in berries, grapes, and wine, while flavonols are found in onions, kale, broccoli, apples, and tea (57). Berries, onions, kale, and broccoli are recommended daily foods in the Nutritarian diet guidelines, and the greater intake of anthocyanidins and flavonols in the Nutritarian group likely reflects higher intakes of these foods. The significant association between the intake of anthocyanidins and DunedinPACE and the significant interaction show that diet group moderates the association with predicted DunedinPACE values, with predicted DunedinPACE values higher for the SAD diet group at the same intake of anthocyanidins (Figure 2). Although the relationship between alpha-carotene intake and DunedinPACE in our cohort was not significant, the significant association of lutein + zeaxanthin with DunedinPACE in our cohort and significant associations between higher carotenoid EBPs and lower values of several epigenetic clocks observed in the larger TruDiagnostic cohort support the idea that carotenoid intake contributes to slower biological aging. A key property of EBPs is that they often outperform their directly measured plasma counterparts in predicting disease risk (55). This likely reflects the capacity of DNA methylation to capture cumulative biological exposures rather than acute fluctuations, making EBPs well-suited for potentially capturing sustained epigenetic shifts associated with dietary pattern change.

Foods including onions, apples, and tea are sources of flavonols in the Western diet (58). Flavonol intake was significantly different between the SAD and Nutritarian diet groups Similar to that observed with anthocyanidins and lutein + zeaxanthin, the significant association with DunedinPACE and significant interaction indicates that the diet group moderates the association, with predicted DunedinPACE values higher for the SAD diet group at the same flavonol intake (Figure 1).

Flavan-3-ols are the primary flavonoids consumed in typical American diets, making up 78% of flavonoid intake, and tea and apples are the primary sources of flavan-3-ols (59, 60). Flavan-3-ol intake was significantly different between groups. Flavan-3-ols were positively associated with DunedinPACE and group, and the dietary factor-by-group interaction was also significant. Thus, predicted DunedinPACE values are higher for the SAD diet group at the same intake of flavan-3-ols (Figure 1).

We evaluated food folate intake in addition to total folate intake to reflect folate derived from plant foods rather than folic acid from vitamin supplements. Intakes of both total and food folate were significantly greater in the Nutritarian group (Table 1), and both were significantly associated with DunedinPACE. Also, the significant group-by-dietary factor interactions indicate that predicted DunedinPACE values are higher in the SAD diet group at the same level of food folate intake (Figure 1).

The positive direction of the associations between the dietary factors and DunedinPACE was unexpected. In the WHI, fruits, vegetables, and nuts and legumes were negatively associated with DunedinPACE, and we expected associations with carotenoids, flavonoids, and folate to be negative as well (10). However, diet group modified the associations of anthocyanidins, flavonols, flavan-3-ols, flavones, alpha-carotene, lutein+zeaxanthin, total folate, and food folate with DunedinPACE. The dietary factor by group interactions were driven by the SAD group (Table 3). As shown in Figure 2, positive associations were apparent in the SAD group, whereas the associations appeared neutral or negative in the Nutritarian group. As shown in Figure 3, carotene diols showed mostly negative but a few positive associations with epigenetic aging indicators. More research is necessary to determine the influence of carotenoid, flavonoid, and folate intakes on DunedinPACE.

### 4.2 Contributions of dietary patterns, body composition, and nutrients to epigenetic aging

Both groups in our cohort included only women without obesity, however, BMI was significantly lower in the Nutritarian group. In our previous analysis, after adjusting for BMI, the difference in DunedinPACE between the Nutritarian and SAD groups was no longer statistically significant (41). This result and a previous study that found body weight correlated more strongly with DunedinPACE than the second-generation clocks GrimAge and PhenoAge, together imply that DunedinPACE may be especially responsive to epigenetic changes stemming from gain or loss of body weight (61). BMI and waist-to-hip ratio were two of the 19 biomarkers included in the original pace-of-aging model used to generate the DunedinPACE DNA methylation algorithm (6). Although plant-based diets, including the Nutritarian diet, are associated with lower BMI (62, 63), future studies with groups matched for BMI could more clearly distinguish direct effects of dietary patterns or dietary factors from those of BMI.

On the other hand, a recent intervention trial found an increase in DunedinPACE following a one-year nutraceutical intervention including vitamins, minerals, and botanical extracts, despite a small but significant reduction in BMI (12). Altogether, these findings suggest a complex relationship among diet, body composition, and DunedinPACE, in which overall dietary pattern and body weight are significant drivers of DunedinPACE, whereas specific vitamins, minerals, and phytochemicals may have limited influence. These data also support the use of DunedinPACE as a marker of diet and lifestyle habits that promote healthy aging, consistent with recent research suggesting DunedinPACE is more responsive to diet and lifestyle interventions than second-generation clocks. DunedinPACE has been reduced in response to dietary interventions, including caloric restriction, a vegan diet, and a Mediterranean diet (9). Whole diet and lifestyle interventions may have advantages over those targeting specific nutrients and phytochemicals for slowing biological aging.

Whether DunedinPACE is a predictor of cancer risk is not yet known. Faster DunedinPACE values were associated with increased chronic disease risk, including cancer, in a study of U. S. veterans (31). Several biomarkers used to develop the DunedinPACE algorithm are related to cancer development, such as those estimating glycemic control, body fatness, cardiorespiratory fitness, and inflammation. In addition to modulating DNA methylation, antioxidant and anti-inflammatory effects of carotenoids and flavonoids could contribute to a lower DunedinPACE and lower cancer risk (22, 23, 37, 64). A healthful plant-based diet is associated with lower cancer risk (65). A healthful plant-based diet rich in whole, high-fiber plant foods, such as the Nutritarian diet, could reduce DunedinPACE in part through lower body fat, cholesterol, and inflammation (63, 66–69). Related, we previously observed lower levels of an EBP predicting CRP levels in the Nutritarian group, suggesting lower inflammation than the SAD group (41). Folate, through its role in one-carbon metabolism is necessary for DNA synthesis, repair, and methylation. Presumably, folate status could affect epigenetic clocks including DunedinPACE via DNA methylation. Sufficient folate intake is associated with lower cancer risk, whereas insufficient folate intake and high-dose folic acid supplementation have been linked to increased risk. Aberrant DNA methylation is a potential mechanism linking low folate status and cancer (70, 71). Future studies are needed to determine the relationships between nutrients and polyphenols, DunedinPACE, and cancer risk.

Effect sizes in our study were small, with unstandardized beta coefficients ranging from 0.0000046 to 0.001 (Table 3). In comparison, in an analysis of WHI data, beta coefficients on regression models of foods and food components such as fruits, vegetables, and added sugars on DunedinPACE ranged from approximately −0.10 to 0.025 (10).

Significant results for DunedinPACE but no other clocks, and the significant correlation between PhenoAge EAA and GrimAge EAA, but not DunedinPACE, are consistent with previous research. Individual epigenetic age metrics may serve as indicators of distinct aspects of biological aging, with different combinations of CpG sites responsive to different nutrients, polyphenols, exercise, or other exposures (72). In the WHI, scores on healthy eating indices correlated more strongly with DunedinPACE compared to PhenoAge EAA and GrimAge EAA (10). In an analysis of 51 intervention studies, the authors proposed that if only one clock showed a significant change in response to an intervention, but it had large disagreements with other clocks in other studies, it was likely to be a false positive result. In this analysis, DunedinPACE did not disagree with other clocks, but it was the only significant clock in 17.6% of interventions in which it was significant. According to the authors, this finding suggests DunedinPACE could be more sensitive than other clocks to intervention effects or is responsive to specific intervention effects other clocks are not (9). Another study compared associations of 16 markers of biological aging with age-associated outcomes, such as frailty, cognitive function, type 2 diabetes, and cardiovascular risk. Of the DNA methylation-based biomarkers, the strongest associations were observed for DunedinPACE, followed by GrimAge EAA (73).

### 4.3 Limitations

We acknowledge limitations of our study, including a small sample size, a cohort primarily composed of non-obese white women following one of two specific diets, retrospective self-reporting of dietary patterns, and differences in BMI and menopausal status between groups. Our results may not be generalizable to other populations. Although the Nutritarian group reported following the diet for a mean of 11.0 (SD 6.3) years, retrospective dietary pattern data was collected at a single time point. Longitudinal studies would be required to determine temporal associations between dietary patterns or dietary factors and epigenetic aging indicators.

Measurement errors in the intake of individual dietary factors could have resulted from self-reporting of food intake. We were able to objectively estimate carotenoid status using an epigenetic biomarker proxy. Although not significantly different between groups, the carotenoid EBP result suggested higher intake in the Nutritarian group, consistent with FFQ data. In future research, the use of objective biomarkers of nutrient intake, longitudinal studies incorporating FFQs at multiple time points, or intervention studies increasing specific nutrients, carotenoids, or flavonoids could clarify the relationship between these dietary factors and epigenetic aging.

Menopausal status may affect biological aging indicators, though its effects on DunedinPACE specifically have not been reported (74). One study found an inverse relationship between estradiol levels and DunedinPACE in older women (75). In our study, the greater number of postmenopausal participants in the Nutritarian group could have resulted in a greater mean DunedinPACE value in this group, possibly obscuring DunedinPACE-lowering effects of the individual dietary factors or dietary pattern as a whole. We adjusted for BMI and menopausal status in our regression models for this cohort.

Additionally, due to the limited data on supplements, we were unable to analyze the associations for omega-3 and vitamin D supplementation in this cohort, which have been found to influence epigenetic aging (76).

## 5 Conclusion

As DunedinPACE is responsive to healthy diet and lifestyle interventions, changes in DunedinPACE may help track improvements in health from these interventions. Identifying specific food-derived nutrients and phytochemicals, or overall dietary patterns, that influence the methylation changes driving the DunedinPACE indicator, and clarifying the relationship between DunedinPACE and cancer risk could provide insight into the epigenetic mechanisms driving biological aging and inform dietary recommendations for healthy aging. Our findings support a Nutritarian diet, which emphasizes dietary factors associated with lower cancer risk, including carotenoids, flavonoids, and food folate, as a strategy for slowing biological aging as indicated by DunedinPACE.

These findings suggest that overall dietary patterns modify the relationships of food-derived nutrients and phytochemicals with the pace of biological aging, as indicated by DunedinPACE. Intakes of anthocyanidins, flavonols, flavan-3-ols, lutein+zeaxanthin, total folate, and food folate, dietary factors associated with lower risk of cancer, were significantly positively associated with DunedinPACE. In the context of a diet high in vegetables, legumes, nuts, and fruit, the DunedinPACE value associated with an intake of anthocyanidins, flavonols, flavan-3-ols, lutein+zeaxanthin, total folate, and food folate was lower than that in the context of a standard American diet. Future studies investigating how dietary factors and overall dietary patterns influence DunedinPACE, and the relationship between DunedinPACE values and cancer risk could inform dietary guidelines for healthy aging.

## Data Availability

The data described in this manuscript will be made available upon request pending approval by TruDiagnostic, Inc. and the Nutritional Research Foundation. In order to protect the data privacy of the individuals represented in this cohort, individual applications will be reviewed by TruDiagnostic, Inc.

## Footnotes

1
https://github.com/MorganLevineLab/PC-Clocks

2
https://github.com/danbelsky/ DunedinPACE
